# The vulnerabilities of Venezuelan immigrants in Brazil and Colombia from the perspective of Intervention Bioethics^*^

**DOI:** 10.1590/1980-220X-REEUSP-2023-0081en

**Published:** 2023-08-14

**Authors:** John Edinson Velásquez Vargas, Helena Eri Shimizu, Pedro Sadi Monteiro

**Affiliations:** 1 Universidade de Brasília, Faculdade de Ciências da Saúde, Programa de Pós-graduação em Bioética, Brasília, DF, Brazil.

**Keywords:** Emigrants and Immigrants, Bioethics, Humans, Social Vulnerability, Emigrantes e Inmigrantes, Bioética, Humanos, Vulnerabilidad Social, Emigrantes e Imigrantes, Bioética, Humanos, Vulnerabilidade Social

## Abstract

**Objective:**

To understand the vulnerabilities to which Venezuelan immigrants living in Brazil and Colombia are exposed, from the perspective of Intervention Bioethics.

**Method:**

Qualitative study, carried out through a semi-structured interview, with 15 immigrants living in Brasília-Brazil and 20 in Medellín-Colombia, analyzed by the IRAMUTEQ software, in the Descending Hierarchical Classification and Similitude Analysis modalities.

**Results:**

The first thematic axis dealt with the reasons for immigrating, above all, difficulties in accessing food and health services. The second axis revealed the trajectory of the migration process, especially the adversities faced before arriving in the countries. The third axis highlighted the challenges of integration in the destination countries, with emphasis on the processes of exclusion and discrimination faced.

**Conclusion:**

It was observed that both Brazil and Colombia need, as proposed by Bioethics of Intervention, to develop policies to reduce the vulnerabilities of immigrants to guarantee a dignified life without discrimination against them.

## RESUMO

**Objetivo:**

Compreender as vulnerabilidades a que estão expostos os imigrantes venezuelanos radicados no Brasil e na Colômbia, na perspectiva da Bioética de Intervenção.

**Método:**

Estudo qualitativo, realizado por meio de entrevistada semiestruturada, com 15 imigrantes que vivem em Brasília-Brasil e com 20, em Medellín-Colômbia, analisados pelo *software* IRAMUTEQ, nas modalidades Classificação Hierárquica Descendente e Análise de Similitude.

**Resultados:**

O primeiro eixo temático tratou dos motivos para imigrar, sobretudo, as dificuldades de acesso aos alimentos e aos serviços de saúde. O segundo eixo revelou a trajetória do processo migratório, especialmente as adversidades enfrentadas até a chegada aos países. O terceiro eixo evidenciou os desafios da integração nos países de destino, com destaque para os processos de exclusão e discriminação enfrentados.

**Conclusão:**

Observou-se que tanto o Brasil como a Colômbia precisam, conforme propõe a Bioética de Intervenção, desenvolver políticas de redução das vulnerabilidades dos imigrantes para garantir uma vida digna e sem discriminação a eles.

## RESUMEN

**Objetivo:**

Comprender las vulnerabilidades a las que están expuestos los inmigrantes venezolanos que viven en Brasil y Colombia, bajo la perspectiva de la Bioética de la Intervención.

**Método:**

Se trata de un estudio cualitativo, llevado a cabo mediante entrevista semiestructurada, entre 15 inmigrantes que viven en Brasilia, Brasil y entre otros 20 en Medellín, Colombia. Los datos se analizaron con el programa IRAMUTEQ, según las modalidades Clasificación Jerárquica Descendente y Análisis de Similitud.

**Resultados:**

El primer eje temático abordó las razones para emigrar, especialmente las dificultades para acceder a la alimentación y a los servicios sanitarios. El segundo eje reveló la trayectoria del proceso migratorio, principalmente lo relacionado a las adversidades afrontadas hasta la llegada a los países de destino. El tercer eje destacó los retos de la integración en dichos países, haciendo hincapié en los procesos de exclusión y discriminación a los que se enfrentan los inmigrantes.

**Conclusión:**

Se observó que tanto Brasil como Colombia necesitan, conforme la Intervención Bioética, desarrollar políticas para reducir las vulnerabilidades de los inmigrantes con el fin de garantizarles una vida digna y sin discriminación.

## INTRODUCTION

Currently, Venezuela is facing a political, social and economic crisis that has led to a deterioration in the conditions and quality of life of the population. This instability has generated several social disorders, including the violation of human rights^(1)^.

According to the International Organization for Migration (IOM), an estimated 5.6 million Venezuelans have migrated worldwide^(2)^. Their migration began several years ago internally, within the country itself. But, with the passage of time and given the inhuman conditions in which they live, especially related to food, health and education^(3)^, immigration processes have shown an increase with different destinations, with preference for neighboring countries. Thus, migration to Colombia represents 1.7 million Venezuelans, this country being the main destination for migration^(2)^. There are currently 345,000 Venezuelans in Brazil^(3)^.

Among the reasons for this, there are economic determinants as one of the main factors of immigration^(4)^, results of the financial imbalance, due to the drop in international oil prices, Venezuela’s main export product, and the tough sanctions imposed by the United States, which block imports, in addition to inflation, which makes it difficult to acquire items of basic needs^(4)^.

Other situations that Venezuelan immigrants face and that motivate them to leave the country are difficulties in accessing health services and acquiring essential medicines. In addition, the massive departure of health professionals makes providing care more difficult^(5)^.

The same applies to education, as low salary conditions for teaching staff, both in schools and universities, result in a shortage of qualified workforce^(6)^. Given this situation, the constant search for improved individual and family living conditions is the main motivation for Venezuelan migration, which, as mentioned before, experiences various deficiencies in accessing basic needs, as well as domestic gas, clean water, and energy^(4)^.

These situations have led Venezuelans to experience situations of extreme vulnerability. This concept derives from the Latin word “valnus,” which means “wound,” indicating that vulnerability can be understood as the susceptibility to being hurt^(7)^. According to Intervention Bioethics (IB), we are vulnerable when faced with something that takes away our power, which can be another person, an institution, or the State^(8)^. Political and economic interests impose the hierarchization of social relations that lead to exclusion and vulnerability^(8)^. Venezuelan immigrants are victims of persistent situations of discrimination, xenophobia, labor exploitation, and increasing disregard for human dignity^(9)^. These situations make them demonstrate high levels of vulnerability, making migration relevant from a political, social, and economic standpoint^(10)^.

It is within this perspective that the migratory phenomenon needs to be analyzed through the lens of Intervention Bioethics (IB), an anti-hegemonic, broadened, and politicized epistemological proposal that considers the social dimension and aims to contribute to the fight against social exclusion, particularly in defense of the most vulnerable individuals, in the pursuit of social justice and equity. Moreover, from a transdisciplinary perspective, IB allows for an expanded view of the conflicting issues experienced by vulnerable individuals, such as Venezuelan immigrants^(11)^. In IB, vulnerability is a key principle of bioethics as it establishes a dialogue among different perspectives, promoting actions that facilitate the overcoming of conditions that keep immigrants in a vulnerable situation in relation to others^(11)^.

This study aims to understand the vulnerabilities faced by Venezuelan immigrants residing in Brazil and Colombia from the perspective of Intervention Bioethics.

## METHOD

A qualitative approach was used, which allows for understanding, interpreting, and engaging in dialogue with the experiences and lived realities of Venezuelan immigrants within the context of their collective history. This approach enables us to explore their subjectivities, which are influenced and shaped by the culture of the group in which they are embedded^(12)^.

### Data Collection

The study was conducted in the cities of Brasília, Brazil, and Medellín, Colombia, which have received a significant number of Venezuelan immigrants. To select participants, a snowball sampling technique was used. This technique involves identifying an individual who exhibits the characteristics under study and establishing a relationship with other individuals in similar situations, requesting participant referrals for new informants^(13)^.

The following inclusion criteria were considered when selecting participants:

– Venezuelan individuals aged 18 years or older, of both sexes.
– Venezuelan immigrants who have been residing in Brasília and Medellín for at least six months.
– Venezuelan individuals with adequate physical and mental conditions to participate in the interviews.

The study will take into account the following exclusion criteria:

– Individuals with communication difficulties.
– Individuals with terminal illnesses or bedridden individuals.

A semi-structured interview guide was used. Contacts with Venezuelan individuals in Medellín and Brasília were established through phone calls, emails, and WhatsApp. The interviews were conducted in the participants’ own contexts, both at their workplace and at their homes.

In Brazil, a total of 15 individuals were interviewed, including 6 men and 9 women. In Colombia, 20 individuals were interviewed, including 9 men and 11 women. This resulted in a total of 35 interviews. The interviews were concluded once information saturation was reached, determining the final set of participants^(14)^. The interviews took place in the first semester of 2022.

### Data Analysis

The data were processed using the software IRAMUTEQ (Interface de R pour les Analyses Multidimensionnelles de Textes et de Questionnaires) version 0.7 alpha 2^(15)^. For this study, a Descending Hierarchical Classification (DHC) was performed, which highlights the most frequent words and their associations in word classes, allowing for the identification of common themes. The classes generated from the DHC represent the context of meaning of the words and can demonstrate the representations or elements of representations^(16)^.

Additionally, a similarity analysis was conducted to identify the co-occurrence of words mentioned by the interviewees. The results of this analysis reveal the connections between the words and identify patterns or structures of representation^(15)^.

The theoretical framework chosen for data analysis was Bioethics of Intervention (BI), which is committed to discussing the social inclusion of individuals in vulnerable situations, along with equality and justice as references for ethical discussions. The aim is to achieve equity in addressing the ethical issues at hand^(10)^. In BI, vulnerability occurs when power is taken away (by another person, an institution, or the state). The category of alterity allows us to understand the other, considering the principles of responsibility and solidarity^(10,11)^. It is from this perspective that the situations of vulnerability faced by Venezuelan immigrants will be analyzed, as well as the strategies for ensuring dignity and human rights^(10,11)^.

### Ethical Aspects

The study complied with the ethical principles described in resolutions 466/12 and 510/16, and informed consent was obtained for data collection. The research was approved by the Health Research Ethics Committee of the University of Brasília, under protocol number 5.164.716 in 2021, for data collected in Brazil, and by the Research Ethics Committee of the University of Antioquia under protocol number 22-43-971 in 2022 for data collected in Colombia.

## RESULTS

In terms of participants’ profiles, it was observed that the majority of participants, both in Brazil and Colombia, were women. The prevalent age range was between 25 and 35 years, and the participants were mostly single. The educational level varied, with participants having technical or higher education degrees. In Colombia, the majority of immigrants did not have children, while in Brazil, most had children. All participants, from both countries, had regularized their legal status. The predominant religion among the participants was Catholicism, according to Table 1.

**Table 1:** Sociodemographic characteristics of the participants – Brasília, DF, Brazil, 2022; Medellín, Antioquia, Colombia, 2022.

		Colombia n:20	Brazil n:15	Total
*Sex*	Female	11	9	20
	Male	9	6	15
*Age*	Under 25	6	4	10
	Between 25 and 35	8	7	15
	Between 35 and 45	4	3	7
	Over 45	2	1	3
*Marital status*	Single	10	7	17
	Married	2	6	8
	Free union	5	2	7
	Separate	3	0	3
*Education level*	High school	0	8	8
	Technician	10	5	15
	University	9	2	11
	Postgraduate	1	0	1
*Have children*	Yes	8	11	19
	No	12	4	16
*Legal status*	Legal	20	15	35
*Religion*	Catholic	14	11	25
	Christian	1	3	4
	Other	5	1	6

The corpus, consisting of all transcribed interviews from Brazil and Colombia, had an 81.51% utilization rate according to the IRAMUTEQ software, which is considered satisfactory.

**Figure 1:**
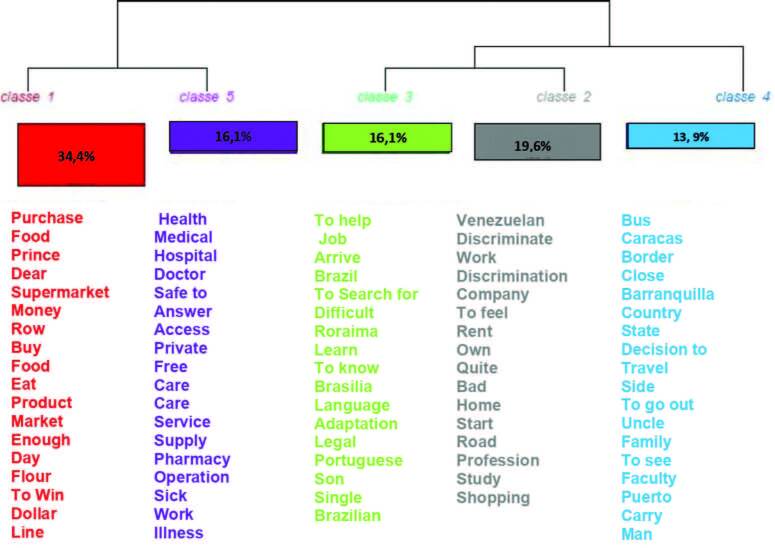
Dendrogram of interviews from Colombia and Brazil.

The dendrogram in Figure 1 summarizes the highlighted thematic axes and word classes. The first thematic axis includes classes 1 and 5, which address the factors that motivated immigration. Class 1 (34.4%) focuses on difficulties accessing food, while class 5 (16.1%) discusses barriers to accessing healthcare services. The second thematic axis reveals the trajectory of the immigration process, with class 4 (13.9%) depicting the paths taken and the adversities faced at the borders. The third and final axis highlights the challenges of integration in the host countries, with class 3 (19.6%) illustrating the obstacles encountered upon arrival, and class 2 (16.1%) describing the processes of exclusion and discrimination experienced in these countries.

From the first thematic axis, Class 1 deals with difficulties in accessing food. The interviews bring to light the situation of hunger felt by the families. The high cost of food, the long queues to access it and the illegal dollarization in Venezuela’s informal trade in order to access essential items were relevant in the participants’ statements. The interviewees also showed that immigration, both to Brazil and Colombia, made it possible to overcome this problem due to the sufficiency of food and the possibility of buying products to meet their needs. However, the memories of family members who are still in Venezuela possibly experiencing food needs were recurrent.

Class 5 describes the barriers to accessing health services in Venezuela and how this situation was part of the motivations to immigrate. The lack of qualified personnel for care, medical supplies, medicines, added to the dollarization, made medical assistance in Venezuela deficient, precarious and high cost.

The interviewees reported that, in both countries: Brazil and Colombia, access to health services is good, with quality care, in addition to finding the necessary medicines for any transitory illness they had, such as headache, flu, among others.

The only difference found was in Brazil, which presented some situations of discrimination due to language, since health professionals did not understand Spanish well.

Axis 2 contains class 4, which describes how the migration process was, the paths taken, the difficulties experienced at the borders, the high costs of tickets, the long bus journeys to reach the destination countries. Regarding the migratory process, a difference that deserves to be highlighted is the way in which some of the Venezuelan migrants who were at the border, more specifically, arrived in Brazil, through the help of the international NGO Caritas, together with the government of Brazil and the forces armed forces, in the Acolhida project.

Axis 3 contains class 3, which describes the adaptation process in Brazil and Colombia. The most relevant point found in the interviews was the search for a job in both countries, as it ensures economic resources to ensure a good quality of life. This job search for immigrants, both from Brazil and Colombia, was not easy. In Brazil, it is worth mentioning the difficulties related to the language, which were important limitations to finding a job and, therefore, made Brazil a more challenging country for Venezuelan migrants.

Class 2 describes the integration process in the destination country. His arrival represented for most participants a change in their way of life, especially to ensure a quality of life that satisfies their needs. They reported that the help received – such as housing, food, clothing donations, among others, as part of the welcome and reception by Brazilians and Colombians – was fundamental to help overcome the conditions of vulnerability.

However, among the main relevance found in the interviews, from both countries, were related to situations of discrimination and xenophobia experienced by Venezuelan migrants. These discriminatory situations were perceived when looking for a job, accessing health services, on the street, among others.

**Figure 2:**
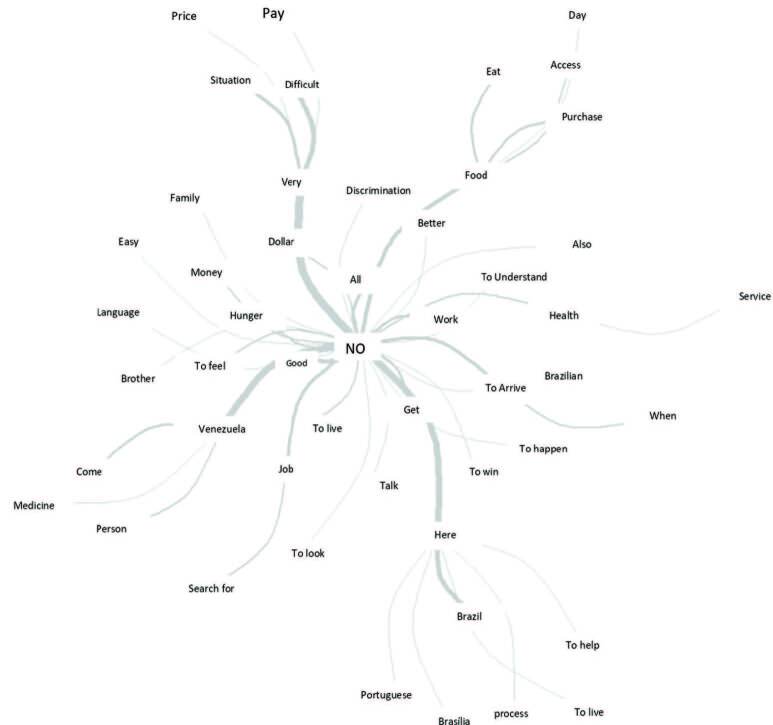
Similarity analysis from interviews with immigrants from Venezuela, in Brasilia-Brazil.

Based on the similarity analysis carried out in interviews with Venezuelan immigrants in Brasilia, Brazil, the terms and their respective connections between them can be identified. In Figure 2, it is observed that there was a semantics of more frequent words: “no” is the central term, followed by the words: “Venezuela”, “very”, “food”, “arrive”, “here”.

The branches of words explain the reasons for leaving the country, especially hunger, lack of money, lack of jobs and the high dollar, which did not allow access to basic goods for the person and his family to live on. Other branches of words show the possibilities represented when they managed to arrive in Brazil, which, despite the language barrier, had help in the housing process in Brasilia/Brazil. It is observed that the words highlight access to work, health services and food. However, they show the process of discrimination experienced.

**Figure 3:**
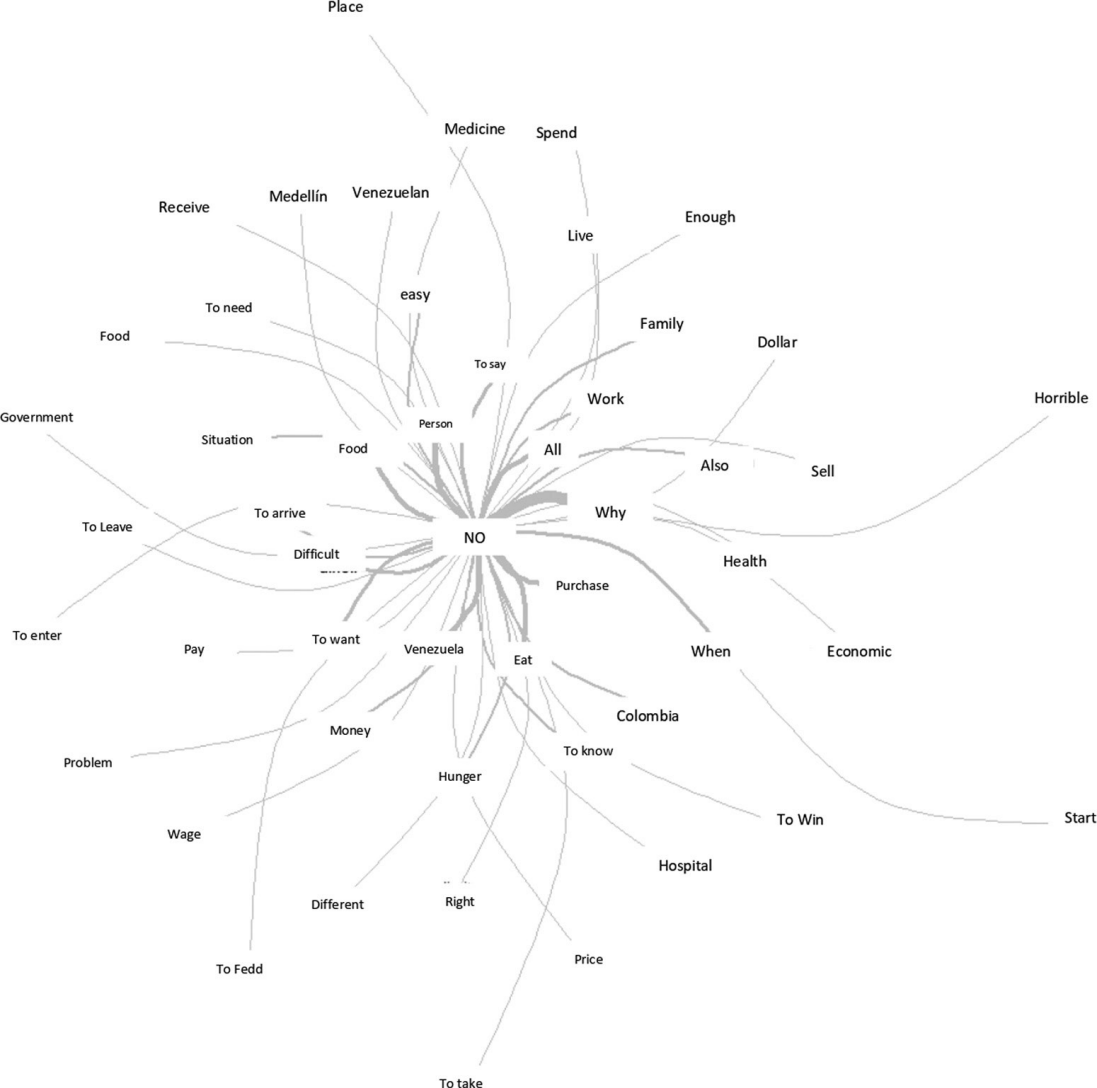
Similarity analysis from interviews with immigrants from Venezuela, in Medellín-Colombia.

Regarding the analysis of interviews with Venezuelan immigrants in Medellín-Colombia, Figure 3 shows the centrality of the words “no” and “why”. These are similar justifications for leaving the country for Brazil. The terms reveal the difficulties of entering the country, which are regulated by the government. In addition, they show the difficulties in accessing health services, because they are private (health, economic, horrible, buy, hospital). The terms also show the possibilities of access to food, work, housing, medication for the person and his family.

## DISCUSSION

It was found that Venezuelan immigrants were exposed to extremely vulnerable conditions in their country. As a result of this scenario, the impossibility of a dignified and quality life conditioned them to immigration; a cyclical and pendular movement, be it temporary or permanent immigration, placed people on the move in a situation of dependence on the receiving countries and, consequently, in a state of vulnerability and significant loss of autonomy^(17)^.

In this sense, it is important to highlight that Venezuelans revealed that they were decisive in the decision-making process for immigration: the difficulties in accessing basic foodstuffs, for various reasons, such as superinflation in prices, shortages or rationing of food, which compromised the human dignity, given the need to satisfy their biological, cultural and social needs and, above all, to avoid food insecurity^(18)^.

Some surveys have shown hunger as one of the main motivations for immigration, followed by inflation, shortages of basic products, lack of jobs, medication, among other social situations^(19)^. In this sense, the World Food Program (WFP) confirmed, at the beginning of 2020, that around 2.3 million of the Venezuelan population live under food insecurity, noting that 55% of households were in a situation of severe or moderate food insecurity^(20)^.

IB recognizes that the vulnerabilities experienced by these immigrants are characterized as persistent situations that require State action, through the construction of equity policies that guarantee respect for human dignity^(11)^. Equity presupposes the need to recognize the equality of rights of each subject based on their differences^(21)^. In this case, the application of the principle of justice implies treating differently people who are not equal, so that equality is understood as the desired result of equity^(11)^.

In addition, IB argues that the promotion of human dignity is achieved by promoting the right to adequate food – both in quality and quantity – as an essential condition for the existence of individuals^(22)^. Access to food and nutrition is essential for the promotion and protection of the health of the Venezuelan immigrant population, allowing integration into society with citizenship, which still needs to be guaranteed through equity policies, both in Brazil and in Colombia.

The purpose of actions to combat hunger and food insecurity experienced by Venezuelan immigrants must be guided by the perspective of avoiding damage to human development and aggression to the dignity of people who arrive in a situation of social vulnerability in other countries^(23)^. It should be noted that the persistence of food insecurity in immigrant homes has not been uncommon.

This picture is multifactorial and multicausal, being associated with the violation of many social rights. This is how IB, due to its interdisciplinary nature, suggests that different policies and actions should encompass these rights to enable the integration of these immigrants into society, guaranteeing them the rights from which they are being violated, that is, withdrawn^(23)^.

It was found that the difficulty of accessing health services was also another relevant factor for immigration, as they felt absolutely unprotected in Venezuela. In this sense, immigrants satisfactorily evaluated access to services, both in Colombia, which has a private and subsidized insurance system, and in Brazil, which has a public and universal system. It was observed that it is essential to adopt institutional actions and practices in health services that consider vulnerabilities and meet the specific needs of Venezuelans, in order to guarantee the right to access to health^(24)^.

In a study carried out in a hospital in Roraima, whose objective was to analyze the impact of immigration on care, it was identified that health services were overcrowded, from primary care services to those of greater complexity^(25)^. Another important finding of the study were the language and cultural barriers, since the language represents the link and the patient’s response to clinical actions. It was found that nursing technicians were resistant to the use of Spanish, which made Venezuelan immigrants experience a feeling of frustration, making them even more vulnerable. In the same study, it was also evidenced that many of the immigrants lived, in addition to the situation of social vulnerability, with several diseases^(25)^. The lack of health care for these people is characterized as a violation of human rights^(5)^.

IB proposes in these situations to rationally analyze and criticize human action and offer pragmatic solutions that can be positive from the point of view of the well-being and health of immigrants, making collective health care necessary and the creation of non-discriminatory inclusion actions and structured on ethical bases, in order to achieve effective results^(24)^.

The right to health, as a collective right, depends on the stage of development of the State, the only one that can recognize health as a right, as in the SUS, which can guarantee measures of protection and care for the recovery of health for all people^(26)^. In this way, the health system has the challenge of achieving universal access to quality health care for immigrants^(5)^. IB suggests including in the evaluation of programs and actions – that are already being carried out – the inclusion of the ethical dimension to guarantee comprehensive access to health services^(23)^.

In order to guarantee the right to full and universal access to health services in the countries receiving immigrants, it is necessary to carry out collaboration agreements and joint actions that consolidate policies for integrating immigrants into health services^(24)^. In this sense, IB makes a political claim for the rights and the possibility of fairness, equality and justice for immigrants in terms of access to health services^(27)^.

Considering the right to access to health and the process of inclusion of the immigrant population, the BI brings up the discussion of the so-called four “Ps” for the exercise of an ethical and responsible health practice, especially in situations of vulnerability such as those that are exposed immigrants: prevention, protection, precaution and prudence. The prevention of possible harm and iatrogenic risks in health services; precaution in risk situations that are not duly considered in care; prudence and protection should guide the assistance of health services, considering the social vulnerability present in immigrants^(11)^.

It was observed in this study that the social discrimination experienced by Venezuelans in Brazil and Colombia is also a starting point for the violation of human rights, as it involves the transgression of people’s dignity. This social discrimination occurs when a certain group receives inferior, derogatory and, in many cases, offensive treatment towards others, due to their origin or connection with a certain group of people^(28)^.

BI promotes a discussion on discrimination and stigma, in the sense of preventing these practices from being persistent by dominant groups to the detriment of those less valued within society. This is how he defends diversity, in an anti-hegemonic perspective, in which people can simply live according to their values, beliefs, sexual orientation, culture and nationality, even if this system of values, beliefs and culture disagrees with moral standards. dominant^(29)^.

Thus, it proposes the idea of tolerance and respect for what is different, but, at the same time, which is based on the equality of human beings, recognizing the other in its difference and uniqueness as an equal. It is through the integration of the recognition of the plurality of the other that there is a rupture of the inequalities that exist due to discrimination and stigmatization, since this is the vision that hinders the enjoyment of human rights^(29)^.

The results of this study revealed that the integration of immigrants, in Brazil and Colombia, needs to be improved in order to offer assistance that includes: adequate reception, identification and registration, sanitary inspection, immunization, migratory regulation, among others. Legal advances are also necessary to guarantee the adequate reception of immigrants, as well as their labor, social and cultural insertion. It is also important to form a space for coexistence, which involves both public actions for emergency care and strategies to reduce difficulties of an intercultural nature^(30)^.

As methodological limitations, it is pointed out the fact that the interviews were carried out online due to the context of the covid-19 pandemic, however, an attempt was made to overcome them through the set of methodological procedures adopted, among them, the transcription of the interviews by the researcher himself. The other was the use of the IRAMUEQ software, which allows apprehending the consensual discourses of this group.

From the BI perspective, health professionals who provide care to immigrants need to understand the different vulnerabilities to which Venezuelans are exposed. Therefore, the daily exercises of alterity^(10,11)^ and empathy may contribute to a better reception of daily needs in health services. In addition, there are challenges: the epistemological, methodological and ethical repositioning for the construction of culturally adapted health care^(30)^.

## CONCLUSION

It was found that Venezuelan immigrants had several vulnerabilities, mainly due to difficulties in accessing food and health services. However, they sought to overcome them through the migration process, which also presented several barriers, putting them in a vulnerable situation. In this sense, it was observed that both Brazil and Colombia need, as proposed by Bioethics of Intervention, to act to develop integration policies for these immigrants to guarantee them a dignified life without discrimination.
